# Overlap between maculopapular exanthema and DRESS among cutaneous adverse drug reactions in a dermatology ward (2008-2012)

**DOI:** 10.1186/2045-7022-4-S3-O6

**Published:** 2014-07-18

**Authors:** Miguel Pinto Gouveia, Ana Gameiro, Inês Coutinho, Neide Pereira, Margarida Gonçalo

**Affiliations:** 1 Department of Dermatology, University Hospital of Coimbra, Coimbra, Portugal

## Background

Immune-mediated cutaneous adverse drug reactions (CADR) present under different clinical patterns, some different from the main phenotypes of CADR. Our objective is to characterize manifestations and culprit drugs in CADR that required hospitalization, particularly exanthema associated with few systemic symptoms without fulfilling the European DRESS criteria (MP/DR).

## Methods

A retrospective study evaluated patients hospitalized in a Dermatology ward between 2008-2012. Sex, mean age, culprit drugs, latency period, exanthema characteristics and systemic symptoms were evaluated comparing MP/DR with MPE and DRESS.

## Results

We analyzed 132 patients (85F/47M; mean age of 63.98±17.68 years) with maculopapular exanthema (MPE) (21.2%), DRESS (28.0%), overlap MP/DR (25.8%), Stevens-Johnson syndrome/toxic epidermal necrolysis (12.1%), acute generalized exanthematous pustulosis (8.3%), fixed drug eruption (3.78%) and urticaria/angioedema (0.76%), caused mainly by allopurinol (36.5%), antimicrobials (30.8%) and anticonvulsants (14.4%). MPE occurred in 28 patients, 21F/7M, mean age of 68±19,71 years ; DRESS in 37 patients, 25F/12M, mean age of 61,38±18,07 years, and MP/DR in 34 patients, 13M/21F, mean age of 64.06 ± 17.06 years, with no significant difference between sex or age. In MP/DR, generalized exanthema was associated with face involvement in 73.5% vs 89.2% in DRESS. Liver was affected in 61.8% vs 78.4% in DRESS, with mild cytolysis in 90.5% (median highest ALT value 109 IU/L, Interquartile range (IQR) 75-162) and mild cholestasis in 61.9% (medium highest GGT 196 IU/L, IQR 87-337), values usually inferior to DRESS (median ALT 131 (IQR 92-392) and GGT 211 (IQR 110-519). Eosinophilia occurred in 11.8% of MP/DR vs 78.4% in DRESS, with an average relative eosinophilia of 18.8%±12% vs 16%±7% in DRESS. Allopurinol was the most frequent culprit drug both in MP/DR (38.5%) and DRESS (51.1%), antibiotics were the culprit in 47.5% of MPE, 26.9% of MP/DR and 20.7% of DRESS. Latency period in MP/DR (18.06 ± 13.17 days) was significantly longer than in MPE (11.04 ± 9.33), however shorter than DRESS (23.85 ± 13,80).

## Discussion and conclusion

We identified a MP/DR overlap syndrome with many features suggesting a less severe DRESS, a mini-DRESS, in a continuum spectrum among exanthematous CADR, with MPE and DRESS as the two poles of the spectrum.

